# Cannabis-Induced ST-Segment Elevation Myocardial Infarction With Possible Coronary Artery Dissection: A Case Report

**DOI:** 10.7759/cureus.39594

**Published:** 2023-05-28

**Authors:** Hassaan Arshad, Aliaa Mousa, Bashar Oudah, Tigran Kakhktsyan, Mohammad Abu-Abaa, Richard Kass

**Affiliations:** 1 Internal Medicine Residency Program, Capital Health Regional Medical Center, Trenton, USA; 2 Internal Medicine, Capital Health Regional Medical Center, Trenton, USA; 3 Internal Medicine Residency Program, Eisenhower Medical Center, Rancho Mirage, USA; 4 Cardiology, Capital Health Regional Medical Center, Trenton, USA

**Keywords:** intractable nausea and vomiting, acute st-elevation myocardial infarction, coronary artery dissection, marijuana, cannabis

## Abstract

Spontaneous coronary artery dissection is a rare and commonly underdiagnosed cause of acute coronary syndrome. Here, we report the case of a 36-year-old male patient who presented with an acute onset of left-sided chest pain, preceded by several hours of nausea and vomiting. Past medical history was significant for chronic marijuana use and multiple episodes of nausea and vomiting requiring multiple hospitalizations. Urinary drug screen was positive for cannabinoids only, and electrocardiography revealed an ST-segment elevation myocardial infarction. This was complicated by an episode of ventricular fibrillation that was successfully defibrillated and prompted cardiac catheterization, which revealed a coronary intraluminal filling defect and a segmental lesion, suggestive of coronary dissection. No evidence of atherosclerotic plaque was noticed. Stent placement and thrombectomy were pursued and the patient was stabilized. As cannabinoid use is gaining legality and becoming widespread, this case aims to enhance physicians’ awareness of potentially life-threatening complications of its use.

## Introduction

Cannabis or marijuana is the most commonly used illicit drug worldwide. Although the use of cannabis has been reported to have a low level of toxicity and no association with cardiovascular disease hospitalization and mortality in a large cohort study [1], cardiovascular side effects of marijuana have been reported in more recent studies, including arterial dissection, thrombosis, stroke, vasculitis, vasospasm, myocarditis, pericarditis, postural hypotension, arrhythmias, and acute heart failure [2,3]. These are mediated by CB1 and CB2 receptors in the vasculature [2]. Spontaneous coronary artery dissection (SCAD) has been reported in association with cannabis use even in normal coronary arteries [4,5].

## Case presentation

**Figure 1:**
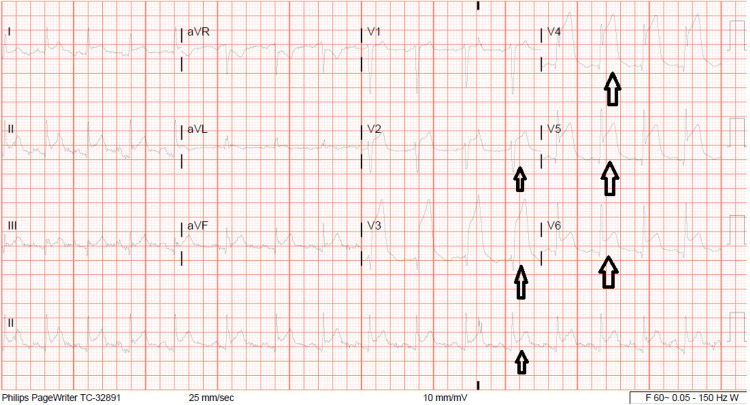
Electrocardiography on presentation showing ST-segment elevation in V1, V2, V3, V4, V5, V6, and lead 2 (arrows).

A 36-year-old male presented to the emergency department (ED) with chief complaints of nausea, vomiting, and chest pain. He reported persistent nausea and vomiting that had started at midnight before the presentation and was followed by a sudden onset of mainly left-sided chest pain in the early morning, which was described as sharp, constant, diffuse, across his chest, and associated with shortness of breath without any reported aggravating or alleviating factors. His past medical history was significant for long-term marijuana use, chronic intractable nausea, and vomiting which led to frequent hospitalizations. Family history was not significant for atherosclerotic disease, including coronary artery disease (CAD), stroke, and peripheral arterial disease. No history of hypertension, hyperlipidemia, or diabetes was reported. In the ED, the patient’s initial vital signs were noted to be a blood pressure of 120/80 mmHg, heart rate of 120 beats per minute in a sinus rhythm, and respiratory rate of 15 cycles per minute with oxygen saturation (SpO_2_) of 97%. At the time of the first presentation, he was fully oriented and in acute distress. No evidence of arthritis, Marfanoid habitus, skin lesions/abnormality, or intravenous injection marks were seen. Laboratory tests were significant for a deranged complete blood count showing a white blood cell count of 25 × 10^3^ cells/µL, hemoglobin of 12.3 g/dL, and platelets of 359 × 10^3^ cells/µL. Urinary drug screen was positive only for cannabinoids and negative for cocaine. A complete metabolic panel significant for hypernatremia at 151 mmol/L, creatinine of 4.40 mg/dL, and elevated blood urea nitrogen at 67 mg/dL was suggestive of pre-renal azotemia secondary to dehydration. Lipase was 368 U/L, and initial troponin was 41.326 ng/mL. Electrocardiogram (EKG) showed ST-elevation in the chest leads V2-V6 (Figure 1).

**Figure 2:**
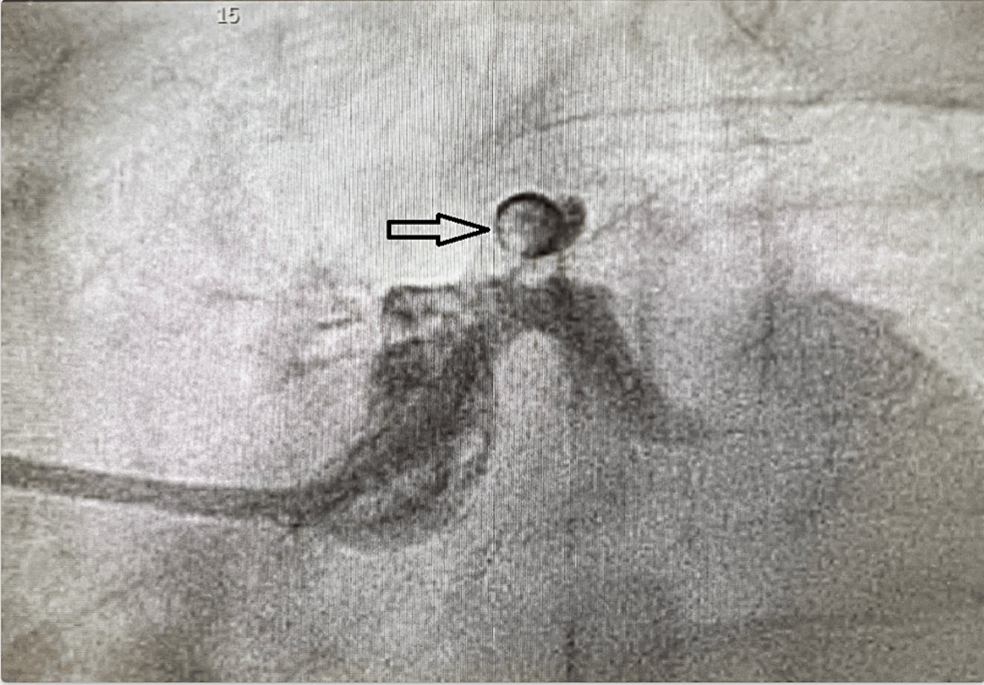
Coronary angiography. Coronary angiography showing a ring of contrast around the proximal left anterior descending artery, suggestive of circumferential coronary dissection rather than a focal plaque rupture event (arrow).

**Figure 3:**
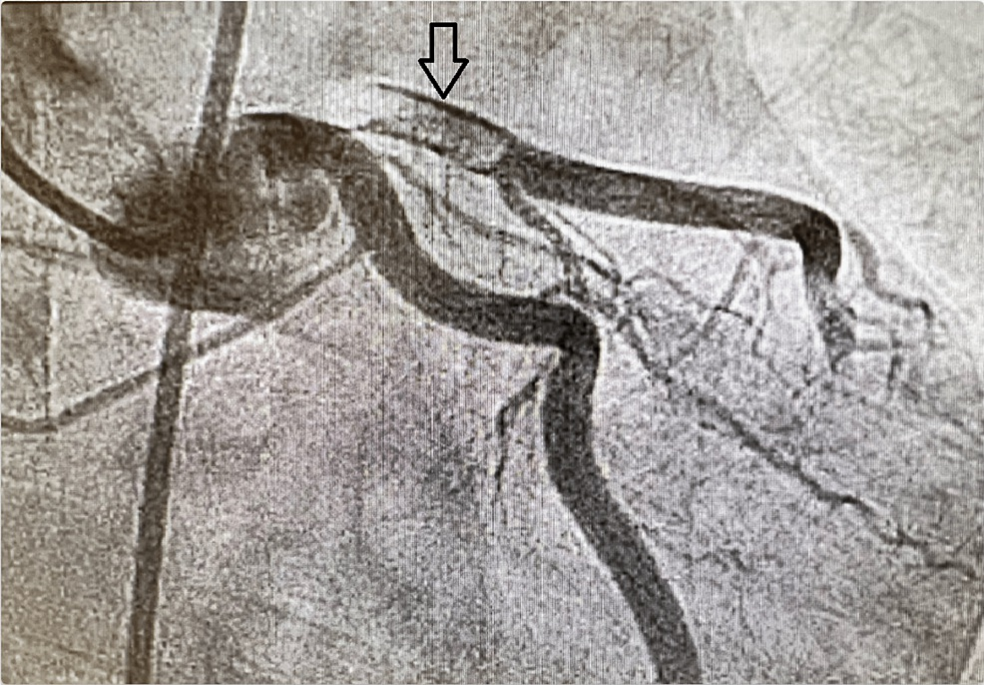
Coronary artery dissection. Coronary angiography showing linear filling of the proximal left anterior descending artery on both sides around a filling defect, suggestive of circumferential dissection rather than a focal plaque rupture event (arrow).

The patient was medicated with nitroglycerine, heparin, and a loading dose of aspirin; however, he continued to report chest pain. He was given fentanyl and was noted to be going into wide complex tachycardia after which the peripheral pulse was not felt. He was immediately defibrillated and returned to sinus rhythm. He was concurrently given amiodarone and magnesium and taken to the catheterization lab (CATH lab). In the CATH lab, the patient underwent coronary angiography which revealed a segmental subtotal lesion at the origin of the left anterior descending artery (LAD) with filling defects suggestive of focal dissection (Figures 2, 3). Successful angioplasty was performed with the introduction of a drug-eluting stent in the proximal LAD with adjunctive extraction thrombectomy of the distal vessel with intravenous integrillin infusion. There was also reduced flow to the distal LAD with persistent occlusion of the apical LAD despite additional treatment. The lipid panel as well as hemoglobin A1c were within normal limits. The thyroid function test was normal as well. However, hemodynamics and symptomatic stability were achieved, and the patient maintained a normal sinus rhythm. A transthoracic echocardiography showed a reduced left ventricular ejection fraction of 30% with LAD territory motion abnormality. The patient was maintained on metoprolol succinate 25 mg daily, valsartan, along with dual antiplatelet therapy with aspirin and clopidogrel.

## Discussion

SCAD is a dissection of a coronary artery that is not traumatic, iatrogenic, or atherosclerotic in etiology [6]. In a large epidemiological study of nonfatal myocardial infarction etiologies, cannabis accounted for only 0.8% of cases compared to cocaine accounting for 20% [7]. Other studies have shown an incidence of 1.1% to 4% [8,9]. In women less than 50 years of age, it accounts for 15-30% of acute coronary syndrome (ACS) cases [10]. The risk of myocardial infarction (MI) is elevated 4.8 times in the first hour after smoking cannabis [11].

SCAD is most commonly reported in young adults, especially in women [6]. It most commonly presents with ACS manifestations. However, some patients present with cardiogenic shock, syncope, or ventricular arrhythmia [12]. Myocardial injury including MI in the case of SCAD usually results from coronary occlusion secondary to intimal disruption and/or intraluminal hematoma formation [6]. Clinical presentation depends on both the extent and severity of the dissection and can range from unstable angina to sudden cardiac death [6]. It is usually misdiagnosed as the suspicion of ACS in young adults, especially females, is low, as well as due to the rarity of the entity [6].

Despite the fact that the exact mechanism of cannabis-induced SCAD is poorly understood, cannabinoids have been reported to increase sympathetic tone which increases the shear stress of the arterial wall, increases blood pressure and heart rate by more than 30%, and induces a dose-dependent severe coronary vasospasm [13]. The most commonly reported mechanism is vasospasm [14]. It also increases carboxyhemoglobin-inducing tissue ischemia, increases oxidative stress disrupting plaques, and increases platelet aggregation and factor 7, thus increasing the risk of thrombosis [5]. The main psychoactive metabolite of cannabinoids, that is, delta-9-tetrahydrocannabinol, is thought to induce the expression of glycoprotein 2B/3A on the platelet surface and induce thrombus formation [15]. In the case presented here, although the angiographic appearance was not typical for coronary artery dissection, one may argue that the typical appearance of false and true lumen of dissection is not a common angiographic finding, hence, the recommendation for topography or intravenous ultrasound if dissection is suspected [13], the confirmed mechanism of myocardial infarction in this case is thrombus formation.

The vast majority of cases of SCAD (70-80%) are seen in females [16,17]. The most commonly affected coronary artery is LAD in 80% of cases, followed by the right coronary artery in only 20% of cases, most of which are seen in males [18]. Multivessel dissection has also been reported in 9-19% of cases [18]. The most commonly reported risk factor is pregnancy, which is attributed to hormonal changes as well as hemodynamic changes including increased cardiac output [16]. Increased estrogen in pregnancy is likely to contribute to the risk as it increases arterial wall susceptibility to dissection [16]. Other reported risk factors include systemic arteriopathies, fibromuscular dysplasia, vasculitis, emotional and physical stress, cocaine use, oral contraceptive pills, and connective tissue diseases including Marfan syndrome and Ehler-Danlos syndrome type 4 [6,16].

Although angiography is required to establish the diagnosis, the typical finding of true and false lumen separated by a line of radiolucency is seen only occasionally and the only finding present might be luminal narrowing, leading to misdiagnosis of atherosclerotic plaque [19]. Other angiographic findings can also include intraluminal filling defects, extraluminal contrast leaks, or spiral dissection [19].

In general, there is no consensus regarding the optimal management of SCAD. The ACS protocol of dual antiplatelet therapy, heparin, and beta-blockers has been shown to maintain true luminal patency in SCAD. However, thrombolytics and coronary angiography are recommended to be avoided due to fear of exacerbation of the dissection, bleeding, and intraluminal hematoma [16,18]. In hemodynamically stable patients with SCAD, a conservative approach is recommended. However, in those with hemodynamic instability, refractory symptoms, or refractory ventricular arrhythmias, the first-line recommended approach is percutaneous coronary intervention (PCI) [18]. However, the use of PCI is challenged by the risk of passing wires into the false lumen, hence, worsening the dissection [19]. A coronary artery bypass graft is preferred in those with failed PCI, unsuitable anatomy, and LAD dissection of proximal dissection [20].

## Conclusions

Cannabis or marijuana may have several adverse cardiovascular complications. SCAD is a rare complication that should always be included in the differential diagnosis of young age adults, especially female patients presenting with ACS. When suspected, coronary angiography is used to confirm the diagnosis. The typical angiographic appearance of true and false lumen or extraluminal contrast leakage is not always present and angiography may reveal only filling defects representing intraluminal hematoma or thrombus formation. Management should be individualized based on clinical status, extent, and severity of the dissection. In general, the ACS protocol of beta-blockers, heparin, and dual antiplatelet therapy should be initiated.
